# Highly suggestive preliminary evidence that the renal interstitium contracts in vivo

**DOI:** 10.14814/phy2.13328

**Published:** 2017-06-23

**Authors:** Omar Flores‐Sandoval, María Eugenia Sánchez‐Briones, Juan F. López‐Rodríguez, Miriam Z. Calvo‐Turrubiartes, Lilia Llamazares‐Azuara, Manuel Rodríguez‐Martínez

**Affiliations:** ^1^ Integrative Physiology LaboratoryDepartment of Physiology and BiophysicsAutonomous University of San Luis PotosíMéxico; ^2^ Renal LaboratoryFaculty of MedicineAutonomous University of San Luis PotosíMéxico

**Keywords:** db‐cAMP, direct interstitial volume expansion, renal interstitial hydrostatic pressure

## Abstract

To learn more about controlling renal interstitial hydrostatic pressure (RIHP), we assessed its response to renal medullary direct interstitial volume expansion (rmDIVE = 100 *μ*L bolus infusion/30 sec). Three experimental series (S) were performed in hydropenic, anesthetized, right‐nephrectomized, acute left renal‐denervated and renal perfusion pressure‐controlled rats randomly assigned to groups in each S. S1: Rats without hormonal clamp were contrasted before and after rmDIVE induced via 0.9% saline solution bolus (SS group) or 2% albumin in SS bolus (2% ALB + SS group). Subcapsular ΔRIHP rose slowly, progressively and similarly in both groups by ~3 mmHg. S2: Rats under hormonal clamp were contrasted before and after sham rmDIVE (time CTR group) and real rmDIVE induced via either SS bolus (SS group) or SS bolus containing the subcutaneous tissue fibroblast relaxant dibutyryl‐cAMP (SS + db‐cAMP group). ΔRIHP showed time, group, and time*group interaction effects with a biphasic response (early: ~1 mmHg; late: ~4 mmHg) in the SS group that was absent in the SS + db‐cAMP group. S3: Two groups of rats (SS and SS + db‐cAMP) under hormonal clamp were contrasted as in S2, producing similar ΔRIHP results to those of S2 but showing a slow, progressive, and indistinct decrease in renal outer medullary blood flow in both groups. These results provide highly suggestive preliminary evidence that the renal interstitium is capable of contracting reactively in vivo in response to rmDIVE with SS and demonstrate that such a response is abolished when db‐cAMP is interstitially and concomitantly infused.

## Introduction

The renal interstitium is the intertubular and intercapillary space occupied by fibroblasts, dendritic cells, a network of collagen fibers, hyaluronic acid (HA), glycoproteins, and interstitial fluid, and is surrounded by a poorly compliant capsule (Lemley and Kriz 1991; Stridh et al. 2012). These components are structured as a gel that supports tubules and capillaries by virtue of its resilience (Tomasek et al. 2002). Even though architecturally the interstitial components are arranged differently in the cortex and the renal medulla (Kaissling et al. 1996), both interstices are mechanically coupled (Garcia‐Estañ and Roman 1989) which does not necessarily mean that they are mechanically identical.

The renal interstitial hydrostatic pressure (RIHP) modulates renal processes such as tubular sodium reabsorption (Granger and Scott 1988), tubuloglomerular feedback (Persson et al. 1982), and pressure‐natriuresis and pressure‐diuresis relationships (Garcia‐Estañ and Roman 1989; Mattson 2003). It determines the renal lymphatic flow (Larson et al. 1984) as well as the release of autacoids into the renal interstitium (Williams et al. 2007; Lieb et al. 2009). In the intact kidney and under hydropenia, the RIHP measured to subcapsular, cortical, cortico‐medullar, or medullar levels by liquid equilibrium techniques is homogeneously positive and approximately 3–5 mmHg (Garcia‐Estañ and Roman 1989; Khraibi et al. 1989). The classical view is that this overall RIHP value is the result of the interplay between the amount of structured and nonstructured interstitial water and the overall elastic properties (Δ*P*/Δ*V*) of the interstitium, including its poorly compliant capsule (Aukland and Reed 1993). The amount of water contained in the interstitium in turn depends on (a) the HA content and its swelling passively restrained by interstitial and capsular collagen network (Hansell et al. 2000), and (b) the dynamic balance among tubular water reabsorption, net transcapillary water flow (Jv) in the cortical and medullary capillaries, and drainage of interstitial water through cortical lymphatics (Wolgast 1985). Because in the intact kidney the increased RIHP consecutive to an increase in renal perfusion pressure (RPP) can occur without changes in hydrostatic and oncotic peritubular cortical capillary pressures, it has been proposed that RIHP is generated in the renal medulla and transmitted to the cortex (Garcia‐Estañ and Roman 1989).

The in vitro phenomenon known as “collagen gel contraction” consists of the ability of fibroblasts in culture to compact a collagen gel to ~10% of its original volume after 24 h when there are optimal concentrations of fibroblasts and collagen (Tomasek et al. 2002). One in vivo representative of this phenomenon is the “cellular subcutaneous tissue contraction,” which determines and modulates interstitial fluid pressure (*P*_IF_) (Berg et al. 2001). Dibutyryl cAMP (db‐cAMP) is a highly permeable lipophilic analog of cAMP that must be hydrolyzed within the cell by esterases to selectively activate the PKA pathway (Schwede et al. 2000), inducing the phosphorylation of myosin light chain kinase and uncoupling the fibroblast's actin‐myosin dynamic filament sliding thereby relaxing the filaments (Ehrlich et al. 1991). It has been reported that db‐cAMP, which increases intracellular cAMP concentration, inhibits both spontaneous collagen gel contraction and cellular subcutaneous tissue contraction (Rodt et al. 1994). Because the prevalence of collagen fibers, interstitial cells, and HA increases toward the renal papilla tip and given that renal medullary interstitial cells in culture show contractile response to endothelin and arginine vasopressin (Hughes et al. 1995), we were interested in investigating whether in the kidney there exists an active physical restriction to the swelling of HA given by the contraction of the medullary interstitial fibroblasts. To determine if this was the case, we first compared the time course of RIHP before and after renal medullary direct interstitial volume expansion (rmDIVE) in hydropenic rats induced by infusing boluses (100 *μ*L in 30 sec) of both isotonic saline solution alone (SS) and 2% albumin in SS. Because there was no difference in the RIHP response with either solution, we then assessed the RIHP response to rmDIVE induced by infusing a bolus of SS in the absence and presence of 5 mmol/L of db‐cAMP administered interstitially and concomitantly. Our hypothesis was that the RIHP response to rmDIVE induced by infusing SS alone should be attenuated by db‐cAMP if fibroblasts reactively contract the renal medullary interstitium.

## Materials and Methods

### Animals

Three series of experiments were carried out in adult male Wistar rats derived from the Charles River 003 strain colony. Their initial body weights were between 250 and 275 g and were housed individually at the animal care facility (with an 12–12 light–dark cycle, and temperature and humidity control) with free access to both a normal sodium rat diet (Rodent diet, Teklad 2018S, Harlan, Maddison, USA) and filtered water and weighed daily in the 10 days leading up to the experimental day. Only those rats that showed an appropriate body weight gain were included in the study. The study was approved by the Faculty of Medicine Internal Committee for the Care and Use of Laboratory Animals and conducted in accordance with the Mexican Guidelines for the Care and Use of Experimental Animals (NOM‐062‐Z00‐1999).

### Surgery

In all series, the rats were anesthetized with sodium pentobarbital (50 mg/kg, i.p., Barbithal NRV, Holland de Mexico, Morelos, México) followed by i.m. administration of atropine (0.05 mg·kg^−1^, Pisa, Guadalajara, México). Once under deep anesthesia, they were placed on a thermostatically controlled table to maintain their body temperature at 37°C and were mechanically ventilated (Rodent Ventilator, Model E683, Harvard Apparatus, Holliston, MA) with a mix of atmospheric air and O_2_ (60 bpm) via an endotracheal cannula (PE‐200) and subjected to both surgical and chemical (10% phenol/absolute alcohol) acute left renal denervation. They were also equipped with (a) a double‐port PE‐50 right femoral vein catheter for infusing solutions and administrating i.v. anesthesia during the surgery, (b) a heparinized PE‐50 left carotid artery catheter for blood sampling, (c) a heparinized PE‐50 left femoral artery catheter which was advanced up to the common iliac artery for renal perfusion pressure (RPP) recording, (d) Roeder knots in superior mesenteric and celiac arteries, (e) a homemade hydraulic aortic occluder placed around the descending aorta above the right renal artery for controlling RPP, and finally (f) a bladder catheter. Next, the right kidney was vascularly excluded and the denervated left kidney was placed dorsal side up into a holder that allowed respiratory movement isolation and avoided ureteral and vascular compression. Once positioned, a homemade, unbeveled, angulate 90°, 31G gauge, 6‐mm‐long stainless steel interstitial catheter connected to a PE‐10 catheter was implanted in the middle of the kidney's greater curvature so that its tip came to the boundary between the outer and inner strip of the outer medulla. In Series 1, this catheter was purged with 2% albumin (ALB) + SS or SS alone, whereas in Series 2 and 3 it was purged with SS alone or SS + db‐cAMP. Once the catheter was in its site a microdrop of cyanoacrylate between the catheter's stabilizer and the kidney capsule avoided leaks. In Series 3, in addition to the interstitial catheter a needle (*θ* = 0.45 mm) Laser‐Doppler probe (Probe 411, Perimed, Järfälla, Sweden) was inserted (incident angle = 52°, introduced 5.5 mm beneath the surface) in the outer medulla using a micromanipulator (Model M3301R, WPI, Sarasota, FL) for recording renal outer medullary blood flow (RoMBF). Once in its site, a fat pad placed at probe insertion point sealed the renal capsule. Finally, a homemade, heparinized, unbeveled, PE‐10 subcapsular catheter was implanted to measure RIHP. Once in its site, a microdrop of cyanoacrylate deposited between the catheter and the kidney capsule avoided leaks. To be included in the analysis, an experiment must have shown a quick RIHP increase in at least 20 mmHg after ~5 sec of renal vein occlusion, followed by a quick return to the baseline after renal vein disocclusion (Garcia‐Estañ and Roman 1989). At the end of the experiment and under deep sodium pentobarbital anesthesia, the left kidney was fixed by aortic anterograde perfusion of 20% buffered paraformaldehyde for verifying the position of both the interstitial catheter (Series 1–3) and the LD probe (Series 3) through the kidney's coarse cross sections.

### Experimental Protocol

#### Series 1. Time course of RIHP in response to renal medullary direct interstitial volume expansion (rmDIVE) induced by both 2% ALB + SS and SS alone

Once the double‐port vein catheter was placed, a 5% glucose (5%G) bolus equivalent to 1% of BW was infused i.v. at a rate of 260 *μ*L/min (~3 mL/15 min) to ensure diuresis. Once the 5%G bolus infusion was finished, the infusion of the maintenance A solution (1% of BSA albumin (ALB, SA‐Ch A‐4503, Toluca, Edo de México) in 0.9% saline solution) was started and held throughout the experiment (57 *μ*L/min). After the surgery, the Roeder knots were run to raise the RPP to ~120 or 130 mmHg, and then the RPP was controlled at 100 mmHg using a homemade manual hydraulic micropump and a baseline (BL) phase of 10 min was instituted. Immediately afterward, rmDIVE was performed by infusing a 100 *μ*L bolus in 30 sec of either 2% ALB in 0.9% saline solution (2% ALB + SS group) or 0.9% saline solution alone (SS group). Next, an experimental (EXP) phase with three periods of 10 min each was instituted.

#### Series 2. Effect of renal medullary interstitial infusion of db‐cAMP on RIHP response to rmDIVE induced by SS

Once the carotid artery was catheterized, a first baseline blood sample (BS1, 150 *μ*L) was taken in preheparinized capillaries for measuring hematocrit (Ht) and plasma protein concentration ([Pr]p). The i.v. infusion of the maintenance A solution was then started and continued throughout the surgery. In order to control for potential neurohumoral influences, the maintenance A solution was substituted at the end of the surgery by the maintenance B solution (hormonal clamp) which consisted of a solution containing aldosterone (66 ng/kg·min, 0.00423 mg/10 mL, Fluka 05521, SA‐Ch), norepinephrine (333 ng/kg·min, 0.025 mg/10 mL, Pridam, Pisa, Guadalajara, Jal., México), vasopressin (0.17 ng/kg·min, 0.0000109 mg/10 mL, SA‐Ch V9879), angiotensin II (AngII) (5 ng/kg·min, 0.00032 mg/10 mL, SA‐Ch A9525), and an ethanolic solution of hydrocortisone acetate (33 *μ*g/kg·min, 2.1 mg/10 mL, SA‐Ch 4126) all of them dissolved in 8 mL of maintenance A solution which continued being i.v. infused at a rate of 57 *μ*L/min throughout the experiment as described by Garcia‐Estañ and Roman (1989). Next, the Roeder knots were run to raise the RPP to ~120 or 130 mmHg, and then the RPP was again controlled at 100 mmHg. Achieved the above, a BL phase of 30 min was instituted and when this ended a second blood sample (BS2, 150 *μ*L) was taken. Approximately 3 min afterward, when all variables had returned to the baseline and according to randomization a sham rmDIVE (time control = CTR group) or a real rmDIVE was performed by infusing a 100 *μ*L bolus in 30 sec of both SS alone (SS group) and SS + 5 mmol/L db‐cAMP (db‐cAMP group). Next, an experimental (EXP) phase of 60 min was instituted and at the end of this phase a third blood sample was taken (BS3, 150 *μ*L).

#### Series 3. Effect of db‐cAMP on RoMBF response to rmDIVE induced by SS

This series was identical to Series 2, with the exception that RoMBF was also measured and that only two groups were studied (SS group and SS + db‐cAMP group).

### Analytical techniques

RPP and RIHP were recorded by coupling precalibrated P23‐XL transducers (Gould Inc, Oxnard, CA) to a 79D Grass polygraph (Grass Instrument Co., Quincy, MA) via Grass 7P1–7DA amplifiers. Both pulsatile waveform signals were electronically attenuated (0.1) and digitized at 1 Hz using a Data Translation DT 2801 analog‐digital board with HP‐Vee software (Hewlett‐Packard Co, Loveland, CO). RoMBF recordings were obtained using a Periflux system (Perimed AB Järflla, Stockholm, Sweden) consisting of a PF 5001 main unit, a PF 5010 LDPM unit with validated electronic linearizer (Nilsson 1984), a probe 411 (0.45 mm *θ*, 0.15 mm fiber separation), and a PF100 calibrator device. The perfusion (arbitrary perfusion units (PU)) and total backscatter signals were continuously recorded and digitized at a frequency of 1 Hz using the same HP‐Vee software. The hematocrit (Ht) was measured by a microhematocrit technique using a microcentrifuge (Clay Adams MP centrifuge, BD Co, NJ; 10,800 *g*/10 min) and a microcapillary reader (Model 2201, International Equipment Company, Boston, MA). Plasma protein concentration ([Pr]p) was measured by refractometry (Clinical Refractometer 5711‐2021, Schuco, ERMA INC, Tokio, Japan) of the plasma derived from the microcapillary Ht. The position of the tip of the renal interstitial catheter in the three series was always localized at the boundary between the outer and inner strip of the outer medulla, whereas the position of the tip of the LD needle probe in Series 3 was always localized in the rostral outer medulla and separate from the interstitial catheter.

### Experimental design and definition of variables

In all series, the experimental protocol was typified as a longitudinal diagnostic subprotocol. Rats were randomly assigned (R software V2.10.1) to groups when rats reached a body weight between 290 and 326 g. Based on the elimination criteria, the eliminated rats were substituted after the first round of experiments by others which were sequentially assigned as they were eliminated (a random process) through a spiral tracking strategy. This continued until the groups were completed. Because the study's main response variable was a difference (ΔRIHP) the study was typified as a two‐way completely randomized cross‐sectional study with a longitudinal diagnostic subprotocol within it. One way being group with two (series 1 and 3) or three levels (series 2) and the other way being time with repeated measurements throughout. In the three series, the RPP was the controlled variable, Ht and [Pr]p were hemodilution indicator variables, and RIHP before, during, and after rmDIVE was the primary measured variable. The RoMBF (PU) before, during, and after rmDIVE was the secondary measured variable in Series 3. The ∆RIHP is equal to EXP RIHP minus the average BL RIHP, and the %∆RoMBF is the % change of RoMBF taken as 100% of the average BL PU. With the exception of Ht and [Pr]p, the remaining variables were measured continuously.

### Statistical analysis

The overall time courses of the variables (RPP, Ht, [Pr]p, RIHP, ΔRIHP, and %∆RoMBF) in the groups were analyzed (95% CI) by a repeated measures MANOVA (RM‐MANOVA) (Weinfurt 2000), with repeated measurements in time factor – before, during, and after rmDIVE – looking for time, group, and/or time*group interaction effects. To assess the effect of time in each group, a RM‐MANOVA by group was performed. In each experiment, the zero time point value taken for the analysis was in the Series 1 the difference between the average of 10 min BL RIHP minus the average of the last minute BL RIHP and in the Series 2 and 3 between the average of the 30 min time point BL RIHP and the average of the 26 min time point BL RIHP. The response variables were modeled by linear fixed models (Regression analysis, 1WANOVA, ANCOVA) and when they did not fulfill parametric statistic criteria, they underwent Box‐Cox transformation (Box and Cox 1964). To minimize the correlation between the mean and variance over time, the %ΔRoMBF raw data underwent log 10 transformation. The best model was chosen on the basis of the highest *r* ^2^ (determination coefficient = explained variation), the lowest Akaike Information Criterion (AIC) (Akaike 1974), and the parsimony principle. Post hoc multiple comparisons were performed by Turkey test, and the model's residuals were tested for normality (Shapiro–Wilk test) and homoscedasticity (Brown–Forsythe test) (Heiberger and Holland 2004). The alpha level imposed was 0.05. Statistical analysis was performed using JMP V10 (SAS Institute, Cary, NC) software and all values are mean ± standard error of the mean.

## Results

### Series 1. Baseline RIHP and time course of ΔRIHP in response to rmDIVE induced by both 2% ALB + SS and SS alone

**Figure 1:**
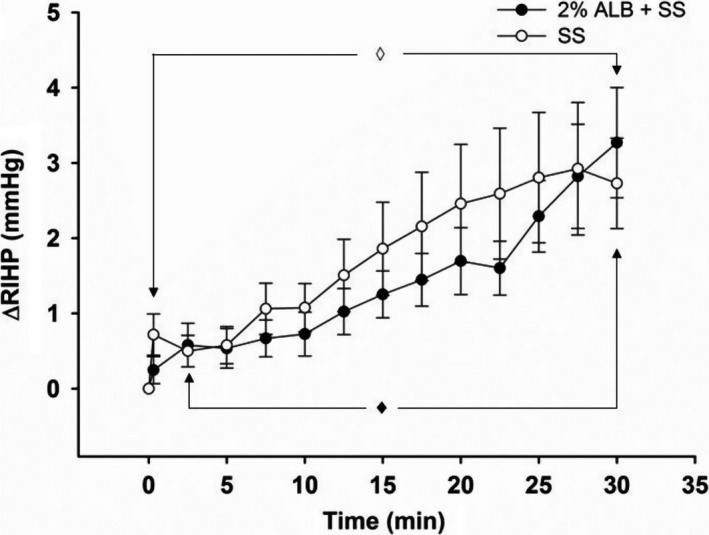
Time course of ΔRIHP in response to rmDIVE in 2% ALB + SS group and SS group in Series 1. 2% ALB + SS group: *n* = 14; SS group: *n* = 15. Each symbol represents the delta of the average of the last 30 sec of each 2.5 min of recording after rmDIVE over the EXP phase minus the average of 10 min of recording before rmDIVE over the BL phase. The overall RM‐MANOVA indicated only time effects (*P* < 0.0001), with no group effect or time * group interaction effect. The RM‐MANOVA by group indicated time effects from the first point (end of rmDIVE = 30 sec) onwards in the SS group (◊), and from the 2.5 min time point onwards in the 2%ALB + SS group (♦).

In this series, the baseline RIHP in the SS group (*n* = 15) was 3.2 ± 0.4 mmHg, whereas in the 2% ALB + SS group (*n* = 14) was 3.1 ± 0.3 mmHg (n.s.). The rmDIVE induced by infusing either SS alone or 2% ALB + SS brought about a slow and progressive increase in ΔRIHP in both groups, reaching maximal values (SS group: 2.7 ± 0.6 mmHg; 2% ALB + SS group: 3.2 ± 0.7 mmHg) ~30 min after rmDIVE (Fig. 1). The overall analysis indicated only time effect (*P* < 0.0001), whereas the group analysis disclosed time effect in the SS group (*P* from 0.01 to 0.003) from the end of rmDIVE onwards and from the 2.5 min time point onwards in the SS + db‐cAMP group (*P* from 0.01 to 0.0002).

### Series 2. Effect of renal medullary interstitial infusion of db‐cAMP on RIHP response to rmDIVE induced by SS

**Figure 2:**
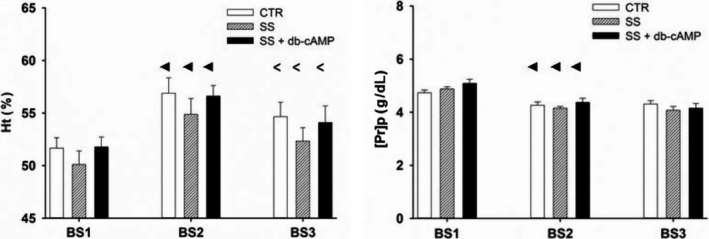
Time course of Ht and [Pr]p) in Series 2. CTR group: *n* = 9; SS group: *n* = 9; SS + db‐cAMP group: *n* = 9. The blood samples were taken at the beginning of the surgery (BS1), before performing the rmDIVE (BS2), and at the end of the experiment (BS3). The overall RM‐MANOVA showed only time effects (*P* < 0.0001), with no group effect or time * group interaction effect.◄ *P* < 0.0001 versus BS1, **<** *P* ≤ 0.0023 versus BS2 by group RM‐MANOVA.

**Figure 3:**
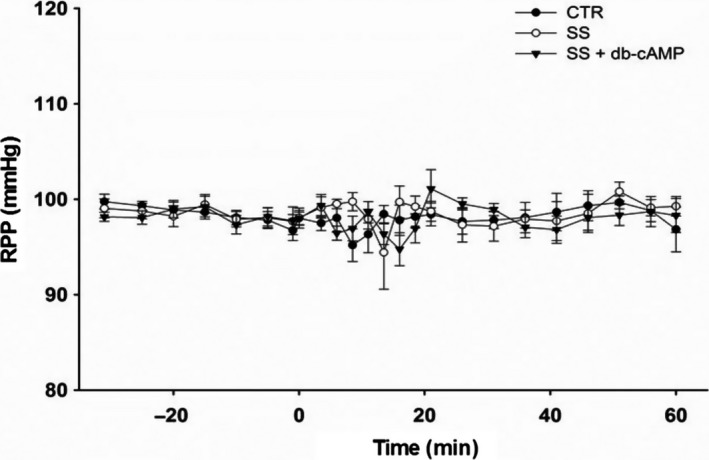
Time course of RPP in Series 2. The symbols represent the average of the last minute of each 5‐min recording during the BL phase and the average of the last minute of each 2.5‐min recording over the first 20 min, and of each 5‐min recording over the next 40 min of the EXP phase. The overall RM‐MANOVA indicated no time, group, or interaction effects.

Because no differences in ΔRIHP response to rmDIVE with both 2% ALB + SS and SS alone were found and because albumin binds cAMP (Arner and Ostman 1975), we evaluate the RIHP response to sham (CTR group, *n* = 9) and real rmDIVE induced by infusing a bolus of SS in the absence (SS group, *n* = 9) and in the presence of 5 mmol/L of db‐cAMP administered interstitially and concomitantly (SS + db‐cAMP group, *n* = 9). The initial body weight in each group was 313 ± 3 g, 309 ± 3 g, and 307 ± 4 g, respectively. The time courses of Ht and [Pr]p are shown in Figure 2. The initial Ht and [Pr]p measured at BS1 did not show differences among groups. The overall analysis indicated only a time effect (*P* < 0.0001). With respect to BS1 values, the Ht increased significantly (*P* < 0.0001) by ~9% in all groups at BS2 and [Pr]p decreased significantly (*P* < 0.0001) by ~12% in all groups at BS2. At the end of the experiment (BS3) 60 min after rmDIVE, the Ht decreased significantly by ~4% with respect to the BS2 value (*P* < 0.0023) and was not different from the BS1 value in all groups. The BS3 [Pr]p values showed no change with respect to BS2 values in any of the groups. The evolution of the RPP throughout the experiment in the three groups is shown in Figure 3. To assess the quality of RPP control at 100 mmHg, the RPP time course was analyzed using the average of the BL phase (1800 samples) and the average of the last minute of recording (60 sec) of every one of the 17 time points in the EXP phase. The overall analysis indicated that there were no time, group, or time* group interaction effects.

**Figure 4:**
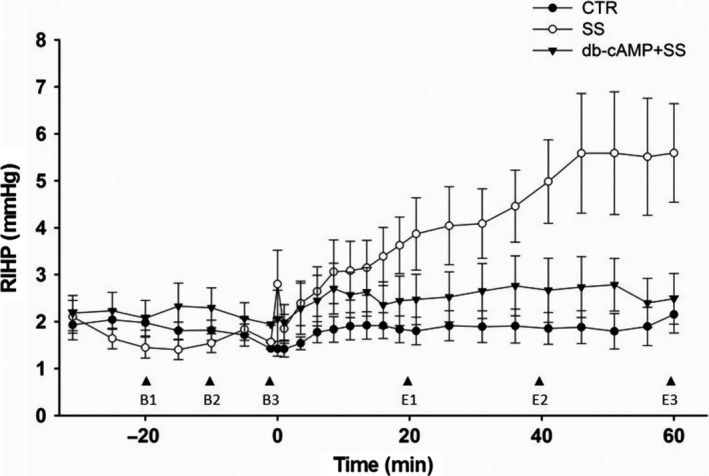
Time course of RIHP in Series 2. During the BL phase, each symbol represents the average of the last minute of every 5 min of recording. During the EXP phase, with the exception of the first symbol which represents the average of the 30 sec of rmDIVE, the remaining symbols represent the average of the last minute of each 2.5‐min recording over the first 20 min and of each 5‐min recording over the next 40 min. Vertical arrow heads (B1, B2, B3, E1, E2, and E3) represent the time points chosen and used for the preliminary bivariate analysis (one‐way ANOVA and ANCOVA).

The time course of RIHP, the primary measured variable, is shown in Figure 4. Each point in the graph represents the average of the last 60 sec of recording for each time point (7 BL and 17 EXP). Preliminary, three BL time points (BL1–BL3) and three EXP time points (E1–E3) were chosen. The analysis indicated that there was no group effect at time point E1 (*P* = 0.0686), but there were at time points E2 (*r* ^2^ = 33%, *P* = 0.0077, 83% power) and E3 (*r* ^2^ = 39%, *P* = 0.003, 92% power). At the last two time points, the Tukey's test showed significant differences (*P* < 0.05) between the SS group and the CTR and SS + db‐cAMP groups, but not between the latter two groups. The potential influence of BL RIHP on EXP RIHP was assessed by bivariate analysis in each group at the selected time points (E1–E3; 9 analyses in total). The fact that in 89% of the cases there was a positive and significant linear association between BL RIHP and EXP RIHP and the fact of not finding differences among groups in the BL RIHP led us to analyze the ΔRIHP instead of absolute RIHP.

**Figure 5:**
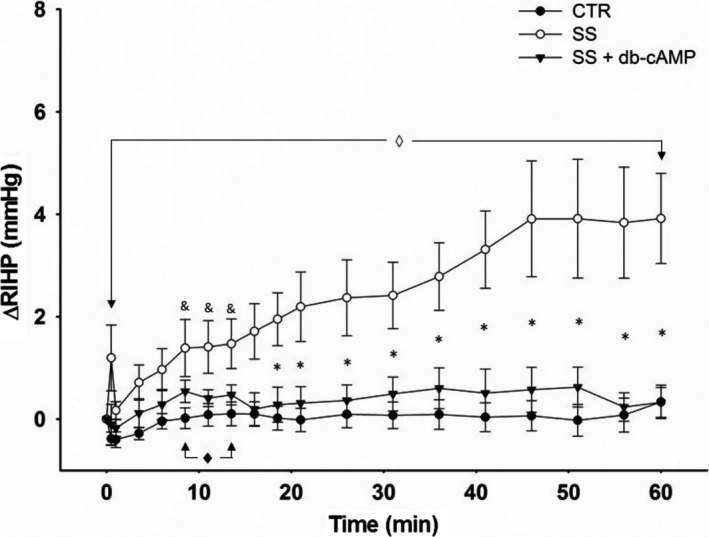
Time course of ΔRIHP in Series 2. Each symbol represents the delta of the average of the last 30 sec of each 2.5 min over the first 20 min and of each 5 min over the next 40 min after rmDIVE during the EXP phase minus the average of 30 min of recording over the BL phase before rmDIVE. The overall RM‐MANOVA indicated time effects (*P* < 0.0001) at all time points as well as group effects (*P* < 0.0014) and time * group interaction effects (*P* < 0.0001). RM‐MANOVA by group: ◊ *P* from 0.03 to 0.0005 versus 0T in SS group; **♦** *P* < 0.02 versus 0T in SS + db‐cAMP group; 1WANOVA + Tukey test: *P from 0.01 to 0.0001 SS group versus SS + db‐cAMP and CTR groups; & *P* from 0.05 to 0.01 SS group versus CTR group.

The time course of ΔRIHP is shown in Figure 5. The overall analysis disclosed time effect (*P* > 0.0001), group effect (*P* < 0.0014), and more importantly a time*group interaction effect (*P* < 0.0001). In the SS group, at the end of the rmDIVE a brief, statistically significant ΔRIHP peak with respect to the zero time point of ΔRIHP was detected (*P* = 0.045). This value was not, however, different from the value reached at the end of the rmDIVE by the other two groups. This little peak (~1 mmHg) in the SS group was followed by a slow and progressive ΔRIHP increase differentiable from the CTR group response from the 8.5 min time point onwards. It reached its maximum value at the 46 min time point (*P* = 0.0008), stabilizing from there up to the 60 min time point (*P* < 0.0005). The effect size for the 60 min time point in the SS group was 4 mmHg, which represented an effect size 14 times higher than that of the CTR group (0.3 mmHg) and 10 times higher than that of the SS + db‐cAMP group (0.4 mmHg). The CTR group showed no changes over time in ΔRIHP and the SS + db‐cAMP group showed no changes over time in ΔRIHP, with the exception of a slight elevation between the 8.5 min and 13.5 min time points which was then indistinguishable from the response showed by the CTR group.

### Series 3. ΔRIHP and %ΔRoMBF time course responses to real rmDIVE induced by both SS and SS + db‐cAMP

**Figure 6:**
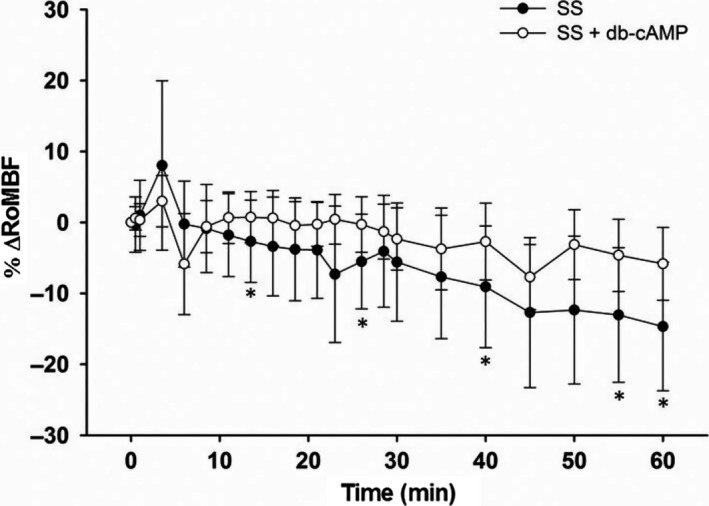
Time course of %ΔRoMBF in Series 3. SS group: *n* = 7; SS + db‐cAMP group: *n* = 7. Each symbol represents the percent change of renal outer medullary blood flow measured in the last 30 sec of each 2.5 min or 5 min over the EXP phase. The overall RM‐MANOVA disclosed only time effects, with no group or time * group interaction effects. The RM‐MANOVA by group showed time effects at the 13.5, 26, 40, 55, and 60 min time points in the SS group, but no time effects in the SS + db‐cAMP group, where only one time point (minute 45) showed a significant decrease with respect to the 0 min time point value. *P < 0.05 versus T0 in SS group; ◊ *P* < 0.05 versus T0 in SS + db‐cAMP group.

The design of this Series was identical to Series 2, with the exception that RoMBF was also measured and only the groups SS (*n* = 7) and SS + db‐cAMP (*n* = 7) were included. The time courses of Ht, [Pr]p and ∆RIHP (biphasic response) were very similar to those observed in Series 2. The overall analysis of %ΔRoMBF showed only time effect (*P* = 0.0013). The analysis by group disclosed time effect in the SS group (*P* = 0.0230) but not in the SS + db‐cAMP group, so that the %ΔRoMBF finished decreasing by 14% in the SS group and by 7% in the SS + db‐cAMP group (Fig. 6). There were no statistically significant differences between the groups at any time point (unpaired tests). To get an idea of the possible association between the ΔRIHP and the %ΔRoMBF, a correlation analysis of these two variables at the 8.5, 13.5, 26, 40, 45, and 60 min time points was performed in each group. These analyses disclosed no association between the two variables at any time point in any of the two groups.

## Discussion

The main findings of this study are (a) there were no differences in the magnitude or in the time course of ΔRIHP when renal medullary DIVE (rmDIVE) was induced by infusing boluses of either 2% ALB + SS or SS alone, (b) rmDIVE induced by infusing a bolus of SS alone brought about a biphasic ΔRIHP response, (c) such a biphasic response was abolished when rmDIVE was induced by infusing a bolus of SS containing 5 mmol/L db‐cAMP, and (d) there were no differences in the overall time course of %ΔRoMBF when rmDIVE was induced by infusing boluses of either SS alone or SS + db‐cAMP.

In the first series the objective was to contrast, in two groups of left renal‐denervated, renal perfusion pressure‐controlled, and hydropenic rats, the ΔRIHP response to rmDIVE induced by infusing a bolus (100 *μ*L/30 sec) of either SS alone or 2% ALB + SS via an acutely implanted medullary interstitial catheter. It is interesting that while we find a slow, progressive, and similar increase in ΔRIHP that reached its maximum ~30 min after rmDIVE in both groups, Granger et al. (1988) and Pawlowska et al. (1988) sharply injected boluses of 50 *μ*L of SS alone and either 2.0% ALB + SS (Granger et al. 1988) or 2.5% ALB + SS (Pawlowska et al. 1988) via a chronically implanted cortico‐medullary matrix and found a differential RIHP response between the SS group (no sustained change in RIHP) and the ALB + SS groups (a significant increase in RIHP that reached its maximum 5 min after completion of the injection and then was stable for the next 30–40 min). Although methodological differences (the method for depositing the bolus: chronically implanted interstitial matrix vs. acutely implanted interstitial catheter; the way of depositing the bolus: injection vs. infusion, the hydration state: mild expansion vs. hydropenia; the control of renal perfusion pressure: without vs. with) could explain these differences, one avenue that must be explored is if the site of deposit of the bolus (cortico‐medullar union vs. medulla) has some influence. While the RIHP results of Granger et al. (1988) suggest that the depuration and retention time of both types of bolus are different when they are deposited in the cortico‐medullary union, the RIHP results of our Series 1 suggest that the depuration and retention time of both types of bolus are similar when they are deposited into the medulla.

The next two series of experiments (2 and 3) were conducted under conditions of hydropenia, renal perfusion pressure control, acute renal denervation, and hormonal clamp. To find similar BL RIHP values among the groups supports the adequate randomization of the experimental units to the groups. The similar Ht increase as well as the similar [Pr]p decrease observed in the groups immediately before rmDIVE (BS2) ensures that the body hydration conditions (hydropenia) in which the animals (and the kidneys) faced the sham or real rmDIVE were similar. The divergent behavior of Ht and [Pr]p at BS2 could be explained by increased protein permeability produced by the i.p. sodium pentobarbital on peritoneal capillaries (Simchon et al. 1990). Because the time courses of the HT and [Pr]p were independent of the studied group, it is believed that these two variables had no influence on the evolution of the ΔRIHP responses (Series 2 & 3) or on the evolution of the %ΔRoMBF responses (Series 3) to rmDIVE throughout the experiment. Our finding that in each group there was a positive association between the BL RIHP values and EXP RIHP values in most of the time points explored (E1, E2 and E3) under the prevailing experimental conditions, and especially under renal perfusion control, is a novel finding that points to the capacity of the renal interstitium for sustaining the BL RIHP differences that exist among rats within each group over time. Furthermore, it indicates that if the EXP RIHP is modified for some reason the modification will be proportional to the individual BL RIHP value of each rat. Thus, to identify with more precision the size effect induced by rmDIVE in each rat and because there were no differences among groups in the BL RIHP, the time course of ΔRIHP was interpreted more than the time course of absolute RIHP. In renal volumetric terms, introducing a bolus of 100 *μ*L into the rat renal medullary interstitium represents, according to Larson et al. (1984), an increase in the size of renal interstitial volume of nearly 50% if this is delivered by injection (sudden distention) or perhaps something smaller and slower if this is delivered by a short infusion (30 sec).

We detected two components of the ΔRIHP response in the SS group. First, at the end of the rmDIVE there was a small and brief ΔRIHP peak that very likely reflects the “passive stress response” given by the interaction between the interstitial volume distension (strain) induced by the SS bolus infusion and the passive elastic properties of the renal interstitium followed by a stress relaxation phenomenon (Junisbekov et al. 2003) typical of viscoelastic tissues such as the kidney (Scola et al. 2012). The second component began immediately after the initial short peak as a slow and progressive increase in ΔRIHP that reached a stable maximum 45 min after the rmDIVE. These two components were also apparent in the SS group of Series 1 without hormonal clamp as in the SS group of Series 3 with hormonal clamp, although the first component was apparently more pronounced in the last. Based on the kinetics shown, the second component appears to be a reactive response rather than a passive response to the rmDIVE induced by infusing the SS bolus. It was hypothesized that if this second component truly constitutes a contractile reactive response of the medullary interstitial fibroblasts to the strain imposed by the rmDIVE, it should be attenuated by concomitantly infusing SS plus db‐cAMP (a well demonstrated subcutaneous tissue fibroblast relaxant) (Rodt et al. 1994). Unexpectedly, we found that the first component of the ΔRIHP response, in essence, disappeared and the second component (with the exception of the first 13 min, where the response showed an attenuated but significant increase with respect to the 0 min time point value) was flat and indistinguishable from the response observed in the CTR group. These findings strongly suggest (1) that the initial “passive stress response” is likely not as “passive” as they have been considered to be so far, and that an active component represented by the state of contraction of the fibroblasts may be participating; (2) that once the renal interstitium shows the “passive stress response” and the subsequent stress relaxation phenomenon, the fibroblasts gradually recover their ability to contract. This might actively restrict the swelling of HA and would explain the time course of the ΔRIHP increase; (3) that db‐cAMP has an extremely short time onset of action compatible with its properties of high permeability and lipophilicity (Schwede et al. 2000), and finally (4) that once db‐cAMP enters the renal medullary interstitium, it has a duration of action of at least 60 min. Together, all this suggests that the renal medullary interstitium has a *sui‐generis*, so far undescribed, mechanical behavior and encourages us to test the effects of anti‐*β*1 integrin antibodies which block the interaction fibroblast – collagen (Reed et al.^1997^) and *α* trinositol, an anti‐inflammatory drug with fibroblast contracting properties (Rodt et al. 1994).

One alternative, but unlikely explanation for the second component of the ΔRIHP response to rmDIVE with SS alone could be the increase in HA content consecutive to the drop in renal medullary interstitial hyperosmolality induced by infusing an isotonic SS bolus. Both in‐vitro (Göransson et al. 2001) and in vivo studies (Hansell et al. 2000; Rügheimer et al. 2009) have documented variable increases in the content of HA in either supernatants or in the renal medulla, in the absence or after the suppression of antidiuretic hormone (ADH) secretion, when the media osmolality or the urinary osmolality have been decreased 100 mOSM or ~1500 and ~1100 mOSM, respectively. Even more, it has been documented (Rügheimer et al. 2008) that ADH, both alone and in conjunction with AngII, reduces the HA content in supernatants of cultured renal medullary fibroblasts and that ADH in vivo reduces renal papillary HA content. Thus, the opposite influence that the dilution and the hormones (ADH and AngII) have on the renal HA content plus the fact that in the present three series (one without and two with hormonal clamp) there was a similar ΔRIHP response to rmDIVE induced by infusing a isotonic SS bolus suggests that the fall in interstitial osmolality in vivo (~250 mOSM) under hydropenic conditions was not of enough magnitude as to trigger an increase in renal medullary HA content in just 30 or 60 min. In the same vein, the idea that the abolition of ΔRIHP response to rmDIVE by SS + db‐cAMP can be explained by an isolated reduction in the renal medullary HA content is seen as even more unlikely because it has been demonstrated in cultures of rat mesangial cells (Parameswaran et al. 1999), retro‐ocular tissue fibroblasts, and adult skin fibroblasts that db‐cAMP increases (Imai et al. 1994) more than decreases the supernatants HA content.

An explanation for ΔRIHP response to rmDIVE in SS and SS + db‐cAMP groups could be that there were differential changes in renal medullary blood flow, decreasing in the SS group and increasing in the SS + db‐cAMP group. The former could cause a lower output of liquid from the renal medullary interstitium with the consequent increase in RIHP, whereas the latter could cause a greater output of liquid from the renal medullary interstitium with the consequent attenuation of the increase in RIHP. This is why it was decided to evaluate the %ΔRoMBF in Series 3 which, apart from reproducing the Ht, [Pr]p, and biphasic ΔRIHP responses to rmDIVE obtained in Series 2, indicated that the insertion of the Laser‐Doppler needle probe by itself did not modify the ΔRIHP response. For the first time, it is reported that rmDIVE induced by infusing a 100 *μ*L bolus of SS in 30 sec brings about a slow, mild, but significant reduction (~14%) in RoMBF which was not differentiable from that induced by infusing SS + db‐cAMP (~7%). Although the experimental design controlled most of the sources of variation, there was a high variability in the %ΔRoMBF values that could have masked small differences between groups. However, the %ΔRoMBF did not show, on average, a frankly divergent behavior between groups, notwithstanding that it has been reported an in‐vitro direct vasodilatory action of cAMP analogs on outer medullary descending vasa recta (Silldorff and Pallone 2001). Furthermore, there was no correlation between the ΔRIHP and the %ΔRoMBF at any time point in any of the two groups. On the whole, this suggests (1) that the elimination of the first component of the ΔRIHP response in the SS + db‐cAMP group when there was still no decrease in %ΔRoMBF was due to a rapid uncoupling action of db‐cAMP on fibroblasts' contractile apparatus; (2) that the abolition of the second component of the ΔRIHP response in the SS + db‐cAMP group was more likely due to an uncoupling action of db‐cAMP on fibroblasts' contractile apparatus rather than a decrease in renal interstitial water content; (3) that in the genesis of the second component of the ΔRIHP response in the SS group the mild decrease in %ΔRoMBF likely played a minor role, if any, and (4) that the differential ΔRIHP response between groups in absence of a differential %ΔRoMBF response between groups plus the lack of association between ΔRIHP and %ΔRoMBF in each group is indicative that both events (ΔRIHP and %ΔRoMBF) happened simultaneously over time but without any relation between them. Series 3 was not designed to scrutinize the mechanisms involved in the reduction in %ΔRoMBF induced by rmDIVE, and neither was it designed to define whether the changes in %ΔRoMBF were the cause or the consequence of the increase in ΔRIHP. Whether the decreases in %ΔRoMBF measured by LD technology represent decreases in erythrocyte velocity rather than real decreases in erythrocyte flow (EF) is still controversial (Evans et al. 2005). Roman and Smits (1986) documented the existence of a strong relationship between erythrocyte flow (EF) measured by the accumulation of ^51^Cr‐labeled erythrocytes (mL/min/100 g) and LD flux signal (LDF = 8.2EF + 8.6, *r* ^2^ = 0.85) in exposed papillae of young and old rats, notwithstanding that the LDF signal was not influenced by changes in erythrocyte concentration within the range (3–5%) of the volume fraction of erythrocytes in rat papilla. Our current view of this scenario is that LDF signal, and therefore our %ΔRoMBF values, are reflecting real changes in renal outer medullary blood flow rather than changes in erythrocyte velocity.

Another alternative explanation for ΔRIHP response to rmDIVE in SS and SS + db‐cAMP groups could be that there were differential changes in urinary sodium and water excretion, increasing in the SS group but increasing further in the SS + db‐cAMP group by a RIHP‐independent, powerful, local, and direct natriuretic effect of the db‐cAMP on thick ascending loop (Bełtowski et al. 2002). This should importantly and significantly reduce the already increased (by the SS bolus) renal interstitial water content. However, such an alternative seems unlikely since Khraibi et al. (1992) have demonstrated in euvolemic rats that the i.v. administration of furosemide results in a significant increase in urinary sodium (9×) and water excretion (10×) and in tubular pressure (~1×) but without any effect on RIHP. Moreover, we did not find differences among groups in Ht or [Pr]p at the end of the experiment (BS3) that suggested a differential natriuresis or diuresis among groups.

## Conclusions

In conclusion, this study conducted in male Wistar rats, under conditions of hydropenia, acute renal denervation, renal perfusion pressure control, and hormonal clamp, demonstrates that the time course of ΔRIHP in response to rmDIVE (induced by infusing a 100 *μ*L bolus in 30 sec) with SS alone is biphasic (early: ~1 mmHg; late: ~4 mmHg) and that is abolished when rmDIVE is induced by infusing a bolus of SS containing 5 mmol/L db‐cAMP. Because above responses were not accompanied by differential changes in the time course of %ΔRoMBF, it provides highly suggestive preliminary evidence that renal medullary interstitium is capable of contracting reactively in vivo in response to the rmDIVE with SS alone. It also demonstrates in hydropenic, acute renal denervated, and renal perfusion pressure‐controlled rats that the time course and magnitude of ΔRIHP in response to rmDIVE with either 2% ALB + SS or SS alone are similar, suggesting that depuration and retention time of both types of bolus are similar when they are deposited into the renal medulla.

## Conflict of Interest

The authors do not perceive any potential conflicts of interest.
